# Supplementary material for: New Species of Mallocybe and Pseudosperma from North China
Source: J Fungi (Basel). 2022 Mar 2;8(3):256. doi: 10.3390/jof8030256 (PMC8949120; doi:10.3390/jof8030256)
Supplement: Supplementary file 1 [file jof-08-00256-s001.zip › Supplementary/Table S1.pdf]

Table S1: Information on sequences used in molecular phylogenetic analyses for *Mallocybe*.

**Species**	**Collection**	**Country**	**GenBank accession number**		
			**ITS**	**LSU**	***rpb2***
Mallocybe africana	BRF4123	Benin	—	MK908842	—
M. africana	HLA0462	Benin	MT458691	MT456364	—
M. africana	MR00369	Burkina Faso	MT476162	MT509361	—
M. africana	MR00385	Togo	MN096194	MN097886	MT465593
M. africana	MR00358	Benin	MT476160	MT509360	MT628398
M. africana	PC 96204	Zambia	—	EU569871	—
M. africana	PC:0088767	Zambia	MN178510	MN178542	—
M. agardhii	JV 7485F	Finland	—	AY380365	AY333772
M. althoffiae	ZT:72/495	Papua New Guinea	NR_163748	EU555444	—
M. arenaria	EL25008	France	FN550937	FN550937	—
M. arthrocystis	PBM 2397	Norway	—	AY380394	AY337402
M. crassivelata	MCVE29561	Slovenia	MN536812	MN537138	—
M. depressa	**BJTC FM1695**	**China**	**OM801899**	**OM801904**	**OM780100**
M. depressa	**BJTC FM1300**	**China**	**OM801895**	**OM801900**	**OM780099**
M. depressa	BJTC C643	China	MW554160	—	—
M. errata	DED8022	—	—	EU569844	—
M. errata	ZT10072	Thailand	—	GQ892936	—
M. errata	ZT 9238	India	—	EU569845	—
M. errata	ZT10108	Thailand	—	GQ892935	—
M. fibrillosa	LVK14390	USA	—	MN178527	MN203518
M. fulvipes	EL8307	Sweden	FN550935	FN550935	—
M. fuscomarginata	BJ890718	Sweden	GU980656	GU980656	—

M. gymnocarpa	SJ980707	Sweden	AM882866	AM882866	—
M. heimii	JV 14932F	Italy	—	AY380379	AY337380
M. isabellina	PERTH:07712758	Australia	MN178501	MN178528	MH618212
M. latispora	JV19640F	Finland	MN178503	MN178529	MN203520
M. leucoblema	PBM1522	USA	—	MN178533	MH577511
M. leucoloma	CLC1869	USA	GU980618	GU980618	—
M. malenconii	JV5498A	Finland	—	EU569870	EU569869
M. multispora	CO4248	USA	MN178509	MN178540	—
M. myriadophylla	JV 19652F	Finland	—	AY700196	AY803751
M. picea	**BJTC FM555**	**China**	**OM801896**	**OM801901**	**OM780096**
M. picea	**BJTC FM569**	**China**	**OM801897**	**OM801903**	**OM780097**
M. picea	**BJTC FM896**	**China**	**OM801898**	**OM801902**	**OM780098**
M. pygmaea	EL48-05	Norway	GU980628	GU980628	—
M. pyrrhopoda	PERTH:08557764	Australia	KP308815	KP170986	KM406226
M. sabulosa	PERTH:07680775	Australia	KP308823	KP170995	KM406236
Mallocybe sp.	ADP060305	USA	MN178513	EU600877	EU600876
Mallocybe sp.	BK 6-June-97-24	USA	—	MN178541	AY337408
Mallocybe sp.	PBM 1922	USA	—	MN178543	MN203527
Mallocybe sp.	PBM 2290	USA	—	EU555446	EU555445
M. siciliana	AMB 18274	Italy	MG757417	MG757419	—
M. squarrosoannulata	SJ84030	Sweden	—	GU980609	—
M. subdecurrens	REH10168	USA	MH024850	MH024886	MH577503
M. subflavospora	E5880	Australia	—	AY380396	AY337404
M. substraminipes	CLC1731	USA	GU980603	GU980603	—
M. subtilior	OKM 24631	Australia	—	AY380398	AY337406
M. terrigena	JV 16431	Sweden	—	AY380401	AY333309

M. tomentosula	PBM4138	USA	MG773814	MK421969	MH577506
M. unicolor	TENN: 06355	USA	MN178525	MN178554	MN203534
M. velutina	MSM#0048	Pakistan	MK990129	MK999927	—
M. velutina	MSM#0049	Pakistan	MK990130	MK999928	—
M. velutina	MSM#0050	Pakistan	MK990131	MK999929	—
Pseudosperma breviterincarnatum	PBM1914	USA	JQ408750	JQ408750	JQ846465
P. breviterincarnatum	BK28080407	USA	EU555451	EU555451	EU555450
P. triaciculare	MSM#0039	Pakistan	MG742423	MG742424	—
P. triaciculare	MSM#0041	Pakistan	MG742429	MG742430	
