# Supplementary material for: New Species of Mallocybe and Pseudosperma from North China
Source: J Fungi (Basel). 2022 Mar 2;8(3):256. doi: 10.3390/jof8030256 (PMC8949120; doi:10.3390/jof8030256)
Supplement: Supplementary file 1 [file jof-08-00256-s001.zip › Supplementary/Table S2.pdf]

Table S2: Information on sequences used in molecular phylogenetic analyses for *Pseudosperma*.

Species	Collection	Country	GenBank accession number	
			ITS	LSU
Mallocybe africana	HLA0462	Benin	MT458691	MT456364
M. africana	MR00369	Burkina Faso	MT476162	MT509361
M. velutina	MSM # 0048	Pakistan	MK990129	MK999927
M. velutina	MSM # 0049	Pakistan	MK990130	MK999928
Pseudosperma aestivum	BK18089706	USA	EU600847	EU600847
P. arenicola	RC GB99-014	France	FJ904134	FJ904134
P. amabile	BAN369	Germany	MW010031	—
P. amabile	BAN3011	Germany	MW010035	—
P. amoris	BAN2931	Germany	MW010038	—
P. amoris	BAN3013	Germany	MW010037	—
P. araneosum	PBM3755	Australia	KJ729878	KJ729904
P. arenicola	EL238-06	France	FJ904133	FJ904133
P. aureocitrinum	BAN2903	Spain	MW010047	—
P. aureocitrinum	BAN260	Germany	MW010041	—
P. breviterincarnatum	BK28080407	USA	EU555451	EU555451
P. breviterincarnatum	PBM1914	USA	JQ408750	JQ319677
P. brunneoumbonatum	MSM#0053	Pakistan	MG742419	MG742420
P. brunneoumbonatum	MSM#00545	Pakistan	MG742421	MG742422
P. brunneosquamulosum	CAL 1308	India	KX073582	KX073586
P. bulbosissimum	EL75-07	Sweden	FJ904160	FJ904160
P. bulbosissimum	EL30-06	Sweden	FJ904158	FJ904158
P. bulbosissimum	**BJTC FM167**	**China**	**OM756770**	**OM756776**

P. bulbosissimum	**HSA 236**	**China**	**OM756772**	—
P. cercocarpi	BK20069807	USA, Utah	JQ319683	JQ319683
P. cercocarpi	UTC: BK255670	USA	MK421964	EU600890
Pseudosperma cf. *flavellum*	GK080924	Great Britain	FJ904129	FJ904129
Pseudosperma cf. *flavellum*	PAM05062502	France	FJ904128	FJ904128
Pseudosperma cf. *flavellum*	EL118-05	Finland	AM882782	AM882782
Pseudosperma cf. *flavellum*	BJ920829	Sweden	AM882774	AM882774
Pseudosperma cf. *microfastigiatum*	EL113-06	Sweden	FJ904156	FJ904156
Pseudosperma cf. *rimosum*	EL71-04	Sweden	AM882786	—
Pseudosperma cf. *rimosum*	JD2008-0241	Great Britain	FJ904125	FJ904125
Pseudosperma cf. *rimosum*	PAM05061101	France	FJ904155	FJ904155
Pseudosperma cf. *rimosum*	JV26578	Estonia	FJ904154	FJ904154
Pseudosperma cf. *rimosum*	EL127-04	Sweden	AM882768	AM882768
Pseudosperma cf. *rimosum*	TAA185135	Estonia	AM882766	AM882766
Pseudosperma cf. *rimosum*	JV22619	Estonia	FJ904157	FJ904157
Pseudosperma cf. *rimosum*	PC080925	Great Britain	FJ904153	FJ904153
Pseudosperma cf. *rimosum*	JV8125	Finland	FJ904152	FJ904152
Pseudosperma cf. *rimosum*	EL81-06	Sweden	FJ904135	FJ904135
P. copriniforme	BAN2800	France	MW010046	—
P. citrinostipes	FYG2909	China	MT072898	MT071203
P. citrinostipes	FYG2903	China	MT072897	MT071202
P. dulcamaroides	EL29-08	USA, Montana	FJ904127	FJ904127
P. dulcamaroides	EL112-06	Sweden	FJ904126	FJ904126
P. dulcamaroides	BAN2924	Sweden	MW010042	—
P. emberizanum	SMNS-STU-F-0901461	Germany	MW647630	—
P. fissuratum	PERTH: E7054	Australia	JQ408770	—

P. flavellum	EL56-08	Sweden	FJ904131	FJ904131
P. flavellum	EL137-05	Sweden	AM882776	AM882776
P. friabile	TENN:068384	USA	MH216095	MH220272
P. gilvum	**BJTC FM1941**	**China**	**OM801910**	**OM801917**
P. gilvum	**BJTC FM1875**	**China**	**OM801911**	**OM801914**
P. gracilissimum	TENN:066946	Australia	KP171123	KJ801179
P. griseorubidum	CAL1253	India	KT180326	KT180327
P. guttuliferum	BAN304	Switzerland	MW010043	—
P. guttuliferum	MCVE 21581	Italy	JF908233	—
P. holoxanthum	ACAD:11683	Canada	MH024853	MH024884
P. hygrophorus	EL97-06	Sweden	FJ904137	FJ904137
P. keralense	TBGT12854	India	KP636861	KP171059
P. keralense	K(M) 191712	India	KM924523	KM924518
P. huginii	SMNS-STU-F-0901564	Austria	NR_173974	MW647628
P. laricis	**BJTC FM887**	**China**	**OM801905**	**OM801912**
P. laricis	**BJTC FM924**	**China**	**OM801907**	**OM801913**
P. luteobrunneum	CAL 1260	India	KX073580	KX073584
P. melliolens	PAM05052303	France	FJ904148	FJ904148
P. melliolens	EL224-06	France	FJ904149	FJ904149
P. mimicum	EBJ961997	Sweden	FJ904124	FJ904124
P. mimicum	TK2004-114	Sweden	AM882781	AM882781
P. napaeanum	BAN2947	Germany	MW010040	—
P. napaeanum	BAN3015	Austria	MW010045	—
P. napaeanum	BAN2948	Germany	MW010044	—
P. neoumbrinellum	HMJAU25742	China	MH047249	MG844977
P. niveivelatum	BK21089714	USA	JQ319695	JQ319695

P. niveivelatum	BK27089718	USA	EU600831	EU600831
P. notodryinum	B12446 (F)	Costa Rica	MK607030	—
P. obsoletum	EL17-04	Sweden	AM882769	AM882769
P. obsoletum	BJ890915	Sweden	AM882770	—
P. obsoletum	WTU:AU10245	Canada	HQ201337	HQ201338
P. occidentale	BK27089703	USA	EU600893	EU600893
P. pakistanense	LAH35285	Pakistan	MG958608	MG958608
P. pakistanense	LAH35283	Pakistan	MG958609	—
P. pakistanense	LAH35285	Pakistan	MF588965	—
P. perlatum	BJ940922	Sweden	AM882772	AM882772
P. perlatum	EL74-04	Sweden	AM882771	AM882771
P. pinophilum	MSM#0046	Pakistan	MG742414	MG742418
P. pinophilum	MSM#0047	Pakistan	MG742417	MG742415
P. pseudoniveivelatum	**BJTC FM1660**	**China**	**OM801909**	**OM801915**
P. pseudoniveivelatum	**BJTC FM1656**	**China**	**OM801906**	**OM801916**
P. rimosum	EL75-05	Sweden	AM882762	AM882762
P. rimosum	PAM06112703	Corsica	FJ904143	FJ904143
P. rimosum	**BJTC FM657**	**China**	**OM756773**	**OM756778**
P. rimosum	**BJTC FM1249**	**China**	**OM801908**	**OM756777**
P. solare	SMNS-STU-F-0901563	Germany	NR_173973	MW647627
P. solare	**BJTC FM1865**	**China**	**OM756774**	**OM756780**
P. sororium	Kuoljok0512	Sweden	FJ904150	FJ904150
P. sororium	JV15200	Sweden	FJ904151	FJ904151
P. sororium	TENN:063504	USA	JQ408781	JQ319705
Pseudosperma sp.	TR138_05	Papua New Guinea	JQ408792	JN975009
Pseudosperma sp.	TR133_05	Papua New Guinea	JQ408791	JQ319709

Pseudosperma sp.	TR104_05	Papua New Guinea	KP636864	JN975011
Pseudosperma sp.	BAN121	Germany	MW009050	—
Pseudosperma sp.	FYG1120	China	MT072906	—
Pseudosperma sp.	PL42609	Australia	KJ729879	KJ729905
Pseudosperma sp.	SX2014092604	China	KR733590	—
Pseudosperma sp.	PBM2601	USA	EU523562	EU600852
Pseudosperma sp.	KR-M-0044827	Germany	MT006032	—
P. squamatum	TK96-109	Sweden	AM882780	AM882780
P. squamatum	PAM05052301	France	FJ904132	FJ904132
P. squamatum	SJ08003	Sweden	FJ904136	FJ904136
P. spurium	SJ92-010	Sweden	AM882785	AM882785
P. spurium	SJ92-017	Sweden	AM882784	AM882784
P. spurium	SM92-013	Sweden	AM882783	AM882783
P. spurium	Stordal18318	Norway	FJ904139	FJ904139
P. spurium	JV2609	Finland	FJ904138	FJ904138
P. triaciculare	MSM#0039	Pakistan	MG742423	MG742424
P. triaciculare	MSM#0041	Pakistan	MG742429	MG742430
P. triaciculare	MSM#0040	Pakistan	MG742426	MG742427
P. umbrinellum	JV17954	Estonia	FJ904166	FJ904166
P. umbrinellum	PC081010	Great Britain	FJ904164	FJ904164
P. vinosistipitatum	ACAD:11758	Canada	MH586781(ITS1)/ MH586818(ITS2)	—
P. xanthocephalum	PAM00100606	France	FJ904130	FJ904130
P. yunnanense	HMJAU25840	China	MH047250	MG844975
